# Supplementary material for: Inside the adolescent voice: A qualitative analysis of the appeal of different tobacco products
Source: Tob Induc Dis. 2021 Feb 26;19:15. doi: 10.18332/tid/132856 (PMC7908110; doi:10.18332/tid/132856)
Supplement: Supplementary file 1 [file TID-19-15-s1.pdf]

Inside the adolescent voice: A qualitative analysis of the appeal of different tobacco products

Jessica Liu[1], Divya Ramamurthi[2], Bonnie Halpern-Felsher[3]

ABSTRACT

INTRODUCTION While cigarette use has decreased, adolescents' overall use of tobacco (e.g. e-cigarettes, cigars, and hookah) has increased. The purpose of this qualitative study is to highlight the decision-making process of adolescents to use certain products over others and why certain tobacco products appeal to them.

METHODS Twenty-five participants were recruited from a larger study surveying adolescents' perceptions and tobacco use (772 high school students). The participants were involved in one-on-one semi-structured phone interviews on the appeal of different tobacco products. Interviews were recorded, transcribed verbatim, and analyzed by identifying relevant codes and themes.

RESULTS Participants for this study had a mean age of 16.4 (SD=1.2) years; over half (14/25) were female. Three major themes emerged from the interviews: 1) social context and circumstances to use, including using and sharing with peers, and reducing boredom; 2) importance of flavors, smell, taste, smoke tricks, and accessibility of products; and 3) misperceptions and misinformation of product risks.

CONCLUSIONS The findings that emerged showed why participants favored certain tobacco products, especially e-cigarettes, over others. The results support areas for future research and practice, and inform how interventions can better address the appeal of different tobacco products to ultimately prevent adolescent use.

AFFILIATION
1 Department of Social and Behavioral Sciences, Harvard T.H. Chan School of Public Health, Boston, United States
2 Stanford Research into the Impact of Tobacco Advertising, Stanford University, Palo Alto, United States
3 Division of Adolescent Medicine, Department of Pediatrics, Stanford University, Palo Alto, United States

CORRESPONDENCE TO
Bonnie Halpern-Felsher. Division of Adolescent Medicine, Department of Pediatrics, Stanford University, 770 Welch Road, Suite 100, Palo Alto, CA 94304, United States. E-mail: bonnie.halpernfelsher@stanford.edu

KEYWORDS
youth, tobacco, smoking, e-cigarettes, flavors

Received: 15 November 2020
Revised: 20 January 2021
Accepted: 26 January 2021

Tob. Induc. Dis. 2021;19(February):15 https://doi.org/10.18332/tid/132856

INTRODUCTION

Although conventional cigarette use has decreased among adolescents, use of alternative tobacco products such as electronic cigarettes (e-cigarettes, vapes), hookah, and cigars, little cigars, and cigarillos has become increasingly popular[1]. This changing landscape stems largely from tobacco companies continuously developing new products and enhancing their marketing strategies that inherently appeal to a younger audience, especially e-cigarettes[2-4]. These alternative tobacco products carry less negative stigma as traditional cigarettes, and adolescents tend to perceive them as harmless[5,6].

Surveys assessing youth perceptions of and reasons for using different emerging tobacco products point to four major influences: flavors, marketing, social influences, and perceived low harm[2,3,6-9]. Youth prefer sweet and fruity flavors as well as mint and menthol, while adults tend to favor menthol or tobacco flavors[10]. Social influences and pressures to appear 'cool' amongst peers during adolescence can result in initiation of tobacco use[11]. Studies on the perceived harm of e-cigarettes showed that youth believe e-cigarettes are not harmful to health or not addictive, especially if they are flavored[12]. Recent findings highlight the harms of adolescent e-cigarette use, including: e-cigarette or vaping product use-associated lung injury (EVALI)[13]; increased risk of future initiation of combustible cigarette use[14]; nicotine's negative impact on adolescent brain development

Published by European Publishing. © 2021 Liu J. et al. This is an Open Access article distributed under the terms of the Creative Commons Attribution 4.0 International License. (https://creativecommons.org/licenses/by/4.0/)

and cardiovascular health[15]; and an increased risk of being diagnosed with COVID-19[16]. Perceived reduced harm is also observed for other alternative tobacco products, for example, youth believe products such as hookah have lower risk than cigarettes or cigars[17]. These misperceptions and misinformation are fueled by tobacco companies' marketing targeted towards youth, as well as social media content sensationalizing various tobacco products[3,18].

Quantitative studies such as those noted above have examined adolescents' attitudes towards and use of various tobacco products[19], but these studies have usually focused only on one tobacco product at a time, using investigator-driven variables and questions. Recent studies have used qualitative data to examine adolescents' own perspectives regarding e-cigarettes to inform youth e-cigarette prevention and cessation efforts[20-22]. However, these studies do not focus on the comparison of different tobacco products, including newer alternative products.

The purpose of this qualitative study is to understand adolescents' perception of the appeal of different tobacco products, including reasons for use/non-use, social influences, and potential misinformation of each product. Understanding personal motivations and how they fit within greater social contexts through the use of narratives can provide an important lens in analyzing adolescents' tobacco perceptions, motivations, and use. Further, qualitative data allow the gathering of information that comes directly from the adolescent population rather than from surveys that are investigator-developed and may not capture the more nuanced and complex reasons for tobacco use.

METHODS

Participants

Participants were recruited from a cohort participating in the Tobacco Perceptions Study, a longitudinal study of adolescents' and young adults' attitudes towards and use of different tobacco products conducted from 2014–2019. The Tobacco Perceptions Study consists of students from both Northern and Southern California who completed consent packets that included the possibility to be interviewed, and who completed the Wave 1 survey (n=772). Twenty-five participants were purposely sampled, based on ever and never use of tobacco products, and recruited to this qualitative study from the larger sample.

Interview guide

The participants were involved in one-on-one semi-structured interviews. Two similar yet separate semi-structured interview protocols were developed, one for self-reported users of any tobacco product and one for self-reported non-users of any tobacco product, in order to tailor the questions to the experiences that participants may be able to describe about tobacco products. The interviews were designed to elicit responses for appeal of each product, decision to choose, and circumstances to use. Participants were asked to describe their experiences with the use of any tobacco, including combustible cigarettes, e-cigarettes (vapes), hookah, and smokeless tobacco, including experiences and knowledge around flavored products. The interviewers asked open-ended questions such as: 'Tell me the story of the first opportunity you had to use a tobacco product' and 'Tell me what you know about flavors' (see Supplementary file Documents A and B for interview guides).

Procedures

Interviews were conducted in 2015, approximately one year before California changed its minimum age sales law to 21 years, and just prior to the significant increase in youth e-cigarette use. Parent consent and adolescent assent for the interviews were obtained during the initial consent process for the entire project. Participants were recruited for this study, the interview was scheduled, and then the interviewer contacted the participant by phone to conduct the interview. Before each interview began, the participants were reminded that their participation was voluntary, the interview could be stopped at any time, and the interview would be recorded. Interviews were transcribed verbatim using a third-party transcription service. Participants received a $25 gift card via mail for their participation. All procedures were approved by the Stanford University Institutional Review Board.

The primary coder (JL), trained in qualitative research methods, used the software Dedoose to identify themes and ideas throughout the interviews that related to the research questions. The software Dedoose, an application for analyzing and organizing qualitative and mixed methods research, was used to analyze data[23]. The primary coder and another member of the research team (BHF) then met through

iterative meetings to discuss and group the common ideas into the main themes of the results.

RESULTS

A summary of the demographic characteristics of the 25 study participants can be found in Table 1. The mean age of the participants was 16.4 years. Of the participants interviewed, 56% (14) were female and 44% (11) were male. The majority of the participants were Latino/a (n=10; 40%), 6 (24%) were White/Caucasian, 5 (20%) were Asian, and 4 (16%) were mixed race/ethnicity. Ten (40%) of the participants reported ever using any tobacco product (users), and 15 (60%) reported never using any tobacco product (non-users). Of the ten users, 8 had used cigarettes, 6 had used cigars/cigarillos, 9 had used e-cigarettes/vapes, 3 had used chewing tobacco, 2 had used hookah, and 1 had used snuff.

Three major themes emerged from the interview analysis concerning why and under what circumstances adolescents use (or do not use) various tobacco products: 1) social context and circumstances to use, including sharing with peers and reducing boredom; 2) importance of flavors, smell, taste, smoke tricks, and convenience of ordering products online or going into smoke shops; and 3) misperceptions and misinformation of product risks. Although we asked about all types of tobacco products, most of the results focused on findings about e-cigarettes, as they were the most popular product of the users (n=9; 90%). We discuss the themes below and refer to Table 2 for illustrative quotes.

Table 1. Characteristics of interview participants (N=25)

Characteristics	n (%)
**Gender**	
Male	11 (44.0)
Female	14 (56.0)
**Age** (years), Mean ± SD	16.4 ± 1.6
**Race**	
Hispanic	10 (40.0)
White	6 (24.0)
Asian	5 (20.0)
Other/mixed	4 (16.0)
**Tobacco use**	
Non-user	15 (60.0)
User	10 (40.0)
**Products used** (n=10)	
Cigarettes	8 (80.0)
Cigars/cigarillos	6 (60.0)
E-cigarettes/vapes	9 (90.0)
Chewing tobacco	3 (30.0)
Hookah	2 (20.0)
Snuff	1 (10.0)

SD: standard deviation.

Social context and circumstances to use

Both users and non-users discussed the social context of tobacco product usage, as a reason for deciding to use tobacco and the product chosen. Users typically used tobacco with friends, and were exposed to new products by peers and friends. Non-users also described how they were sometimes around users, which is how non-users were able to provide insight on some of the products. Users cited boredom in their daily activities and usual social gatherings as reasons for using tobacco. Both users and non-users also referred to the dynamic of social empowerment and positive image associated with the use of some tobacco products.

Sharing with peers

Many users revealed that their main access to tobacco was through their friends who already had the products, and how the social experience of sharing tobacco products was a part of the peer bonding experience. The act of sharing smoking cigarettes in social settings with groups of friends was described as a way of building community bonding (Table 2, Example 1, labeled Ex 1). Users discussed various ways that they would obtain tobacco products solely through their friends, suggesting that there were a select few key peers who have access to these products, rather than everyone individually buying them. This pattern of obtaining tobacco provided insight about social circles of adolescents, and how tobacco use would spread within friends' groups and teams. A user revealed the social dynamics of being on a sports team in high school as being the reason for their initiation of chewing smokeless tobacco (Table 2, Ex 2). However, one user mentioned how obtaining tobacco products, such as e-cigarettes, through peers can have the drawback of lacking the choice in products and flavors they actually use (Table 2, Ex

Table 2. Illustrative quotes

Themes	*Illustrative quotes*
**Social context**	
Sharing with peers	Ex 1. '… the process of kind of like lighting something and then smoking it [cigarette], and like passing it around, as always been kind of like a – it's kind of a relaxing process and kind of like building a sense of community almost just between the people you're surrounded with.' (User)
	Ex 2. 'I played baseball in high school. And like a lot of baseball players kind of chew in general. So it was just something to do while I was out there. … And then after it just became a habit, I guess.' (User)
	Ex 3. '… I usually just do it [e-cigarette] through friends, so it's just whatever they have.' (User)
	Ex 4. 'The first opportunity I had, I was dating someone who was older than me and they had one. They had an e-cigarette and they let me try it and I used that.' (User)
	Ex 5. 'I'll use it [e-cigarette] when I'm inside [friend's] house … I'm not going to do it around [family].' (User)
	Ex 6. 'It was a party at a friend's house … in their backyard … people were just kind of passing it [e-cigarette] around and I was like "Why not …" .' (User)
Social status	Ex 7. 'It was kind of like the cigar thing where like, you know, it made me feel older and more empowered and stuff.' (User)
	Ex 8. 'A lot of people will say that actors look cool because they're smoking [cigars] or things such as that. I was at the time feeling cool about it.' (User)
	Ex 9. 'Most people that I've met all agree that it's cool. I really haven't found anyone that disapproves of it [e-cigarette].' (User)
Boredom	Ex 10. 'Multiple times a day, usually just when I'm driving, keep me busy, because I get bored driving.' [e-cigarette] (User)
	Ex 11. 'Because I guess it's just more fun to just see how they experience it [e-cigarettes] too and it's just something to do, because it's in moments where we're just really bored or there's nothing else to do around there.' (User)
**Product appeal**	
Flavors	Ex 12. 'Oh, I used that all day. I'd just keep it [e-cigarette] with me in my pocket and when I felt like having a taste of something flavor-y or something, I'd just take a hit … when you smell like blue raspberry and green apple, no one is going to question why you smell like that.' (User)
	Ex 13. 'Even if there were tobacco ones I'd still rather have a flavored one. Just 'cause the tobacco taste isn't really necessarily that good and if I wanted to taste tobacco, I'd smoke an actual cigarette instead of using an e-cigarette.' (User)
	Ex 14. 'I just stick to a menthol, minty kind of flavor because it's, I don't know it's kind of refreshing. Like chewing gum but obviously not chewing gum … I like the refreshing taste of it and it's like the coolness of the mintiness, I guess.' [e-cigarette] (User)
	Ex 15. 'I like that I didn't have to always go outside to smoke a cigarette or have to light it or deal with ash and the harsh odor on yourself. Cigarette smoke stays on you and the e-cigarettes don't at all because there's no scent on them really.' (User)
	Ex 16. 'I know that hookah comes in different kinds of flavors and stuff like that ... Because I hear about it from friends.' (Non-user)
	Ex 17. 'I remember this incident ...when he was puffing his oil, vapor thing, it did give off a fruity scent. I don't remember exactly what. I think it was a fruity scent. I know it comes in different flavors.' (Non-user)
	Ex 18. 'I mean I can smell it when they do it [flavored e-cigarettes], and it smells good I guess.' (Non-user)
Relaxing sensation	Ex 19. '… it [cigarette] was kind of a relaxing thing to do. And it was less harsh that smoking marijuana, like, it didn't make me cough as much, so I remember it was just kind of – that was a nicer feeling.' (User)
	Ex 20. '[Cigarettes] made me feel really calm. It didn't do really anything else to me.' (User)
	Ex 21. 'Chewing is, I don't know, just something to do. It's kind of almost relaxing and simple. So I find pleasure in that as well.' (User)
Accessibility and convenience	Ex 22. 'I like how much easier [vape pens] were to use … Compared to a pipe or even a blunt, actually. Because they don't stay hot. They're a lot less dangerous to me. If you drop one.' (User)
	Ex 23. 'I actually bought it [e-cigarette] online. I was not asked for ID or anything … because I was a minor and I couldn't buy them in stores without an ID, I assume. I thought if I bought them online, maybe they wouldn't check my ID and they didn't.' (User)

Continued

Table 2. Continued

**Product appeal**	
	Ex 24. 'I would order stuff for friends online ... So usually they'll get it online just because if you have a debit card or somebody has a debit card and they're like, a minor still, they can still order it online, like, anonymous.' (Non-user)
	Ex 25. 'We actually found a store that would sell [vapes] to someone that didn't have their IDs ... it was kind of cool.' (User)
Smoke tricks	Ex 26. 'Well, one it's really cool to see that much smoke come out of your mouth and I enjoy the flavor, honestly.' [e-cigarettes] (User)
	Ex 27. 'I do enjoy playing with the smoke, because it's kind of fascinating.' [e-cigarettes] (User)
	Ex 28. 'I know that some of them just think it's cool to kind of just like play around with, when they're bored, to try and do different tricks. I guess. ... From what I know, that seems to be the main purpose, at least for the people who I'm pretty close with.' [e-cigarettes] (User)
	Ex 29. 'And there are other cool things, like the clouds they make and, like, they can make little whirlpools and stuff like that.' [e-cigarette] (Non-user)
**Misperceptions and misinformation**	
Misperceived reduced harm	Ex 30. 'I guess after I tried the actual cigarette I felt like I wanted something, but not something harmful, that harmful at least, so I went to the vape store with my friend.' (User)
	Ex 31. '... So I mean I don't know how much similarities it is to cigarettes, because I assume [vape] is less because it's supposed to be healthier, or not healthier but it's supposed to be not as bad for you as cigarettes.' (User)
	Ex 32. 'You just end up knowing because you hear people talk about it [e-cigarettes] ... I just know that it's, I guess it's less harmful than a cigarette, or like tobacco, or something.' (Non-user)
	Ex 33. 'And e-cigarettes, they provide that [nicotine] but in a supposedly harmless vapor.' (Non-user)
	Ex 34. '... I've always found that cigarette users and people who chew and dip it's just really gross to me. ... And it's kind of weird saying because the cigar's pretty much the same thing. But I thought maybe it wasn't, maybe it didn't have as much tobacco or didn't smell as bad.' (User)
Misinformation	Ex 35. 'It had no nicotine at all. It was just water vapor or whatever they put in the e-cigarette. It was no nicotine.' (User)
	Ex 36. 'I feel that there's not as much tobacco in it [cigars] or so I think ...' (User)
	Ex 37. 'I think it was more just like mental, because my mom's a nurse and then the package says smoking harms ... So I know the consequences of smoking, but I was still kind of consciously disregarding that to continue doing it.' (User)

3). In addition to peers and friends, a user discussed how they initiated use and had access to e-cigarettes through an older romantic partner (Table 2, Ex 4).

Users described that due to inability to use products in their own home, they often found themselves using products such as e-cigarettes at the houses of friends (Table 2, Ex 5). Social parties were also revealed as a location where initiation of tobacco product use would happen, and a user discussed how they felt it was more acceptable to try an e-cigarette for the first time in a party setting (Table 2, Ex 6).

Social status

Adolescents perceived using tobacco as a marker of social status and popularity. A user described using tobacco as a sign of social capital, or something that made one feel more mature, like the symbolism of smoking a cigar (Table 2, Ex 7). Users also described how they believed their peers held the idea that tobacco products, such as cigars and e-cigarettes, are 'cool', especially as depicted in movies and media (Table 2, Ex 8 and Ex 9).

Boredom

Users described using products, especially e-cigarettes, as a means to cope with boredom or just have something to do with friends. One participant discussed using e-cigarettes multiple times a day while driving, a passive activity they needed to do in order to occupy themselves but which they found boring (Table 2, Ex 10). Introducing new products with friends while 'hanging out' was mentioned as a

way to deal with boredom (Table 2, Ex 11).

Product appeal

Both users and non-users described the appeal of various tobacco and nicotine products. Most users mentioned the flavor, taste, smell, and relaxing sensation of tobacco products. They also discussed the accessibility of products, shopping online or at stores that did not request identification cards to ascertain if underaged. Some respondents described feeling intimidated when walking into a smoke shop and usually went in with a friend who was more knowledgeable about tobacco. Smoke tricks were mentioned by both users and non-users as appealing. Each of these areas of product appeal are discussed next.

Flavors and smell

Both users and non-users mentioned the flavors in all types of tobacco products, and especially in e-cigarettes as appealing and reason to use the products. Both users and non-users noted that the flavors were not only appealing because of their sweet, fruity, and minty taste, but also because the flavored products smelled good and masked the true smell of tobacco.

A user described the different fruity flavors as something that would more easily hide their use of e-cigarettes (Table 2, Ex 12). A user discussed how they explicitly did not enjoy the tobacco flavor of e-cigarettes (Table 2, Ex 13). A user described the non-fruity mint or menthol flavors as very refreshing and compared them to the appeal of chewing-gum flavors (Table 2, Ex 14). The more pleasant smells of e-cigarettes and cigars, compared to conventional cigarettes, were also appealing to both users and non-users, and one user mentioned how e-cigarettes conveniently did not need to be lit, could be used indoors, and did not have a lingering smell (Table 2, Ex 15).

Non-users also mentioned flavors, knowing that there were many varieties available. A non-user described how they knew that many flavors existed for products such as hookah (Table 2, Ex 16). They spoke about the smell of flavors, often noting how sweet or good the flavors smelled, information that was obtained from being around others who were using flavored e-cigarettes (Table 2, Ex 17 and Ex 18).

Relaxing sensation

The relaxing effect of using all types of tobacco was described positively in many of the interviews of users. Users perceived smoking cigarettes as calming and intentionally used certain tobacco products for this effect as with cigarettes (Table 2, Ex 19 and 20). A user explained the passive engagement of chewing smokeless tobacco as a relaxing action through the simple repetitive musculoskeletal motions (Table 2, Ex 21).

Accessibility and convenience

Both users and non-users discussed how ease of purchase and use, as well as the convenience of products, influenced their choice to use certain tobacco products especially e-cigarettes over others. Users spoke about e-cigarettes being easier to use in different locations, such as in cars or at friends' houses, or were easier to hide and being discreet while using. This ease of use, including the fact that e-cigarettes do not stay hot, contributed to a user's belief in the lower risk of alternative tobacco products such as vapes (Table 2, Ex 22). Further, the availability to purchase online, where adolescents did not need to provide identification of their age, helped with easier access. A user described how it was easier to purchase e-cigarettes online compared to purchasing cigarettes in stores (Table 2, Ex 23). A non-user described how they would help their friends purchase e-cigarettes online and avoid any age verification (Table 2, Ex 24). Finding stores that sold e-cigarettes without checking age identification was 'cool' (Table 2, Ex 25).

Smoke tricks

One appealing aspect of e-cigarettes specifically was the pattern of the actual vapor or 'smoke' that was produced and how this was seen as 'cool' and entertaining. Both users and non-users described how the 'smoke' from e-cigarettes was a novelty experience to play and enjoy with peers (Table 2, Ex 26 and Ex 27). Adolescents have found ways to manipulate the 'smoke' and create tricks, as one user described how smoke tricks were considered a fun way to pass time with peers (Table 2, Ex 28). A non-user described the vapors as clouds and little whirlpools and found them 'cool' (Table 2, Ex 29).

Misperceptions and misinformation

Both users and non-users were asked about their

perceptions of each type of tobacco product. We defined misinformation as false or inaccurate information of messaging, regardless of authorship[24], which differs from misperceptions, defined as false or inaccurate beliefs of the individual. Both misperceptions and misinformation around various tobacco products were motivations that made the product appealing enough to encourage use, as described next.

Reduced harm

In many interviews, both users and non-users perceived e-cigarettes as less harmful than smoking combustible cigarettes and cigars, and some described e-cigarettes as completely harmless. Some users defined chewing smokeless tobacco as less harmful than smoking tobacco, as it was not directly impacting the lungs. A user described how they transitioned from using combustible cigarettes to e-cigarettes as they considered them a less harmful alternative (Table 2, Ex 30). Non-users were able to discuss their perceived reduced harm of e-cigarettes compared to combustible cigarettes just from hearing about it through friends (Table 2, Ex 31). A non-user described e-cigarettes as a method of delivering nicotine through 'harmless vapor' (Table 2, Ex 32). A user used the word 'healthier' to compare vapes with combustible cigarettes (Table 2, Ex 33). Cigars were also perceived to be less harmful than cigarettes, because cigars did not 'smell as bad' (Table 2, Ex 34).

Misinformation

Lack of accurate knowledge around tobacco products emerged as a decision-making factor for both users and non-users. Adolescents have strong assumptions about various tobacco products that may not be true. The perception that e-cigarettes were harmless was influenced by the fact that multiple users stated believing that there was no nicotine at all in e-cigarettes, with one user describing it as 'just water vapor' (Table 2, Ex 35). With cigars, a user said that they 'think' they contain less tobacco (Table 2, Ex 36). However, the simple lack of accurate information regarding harms of tobacco products was not the only motivator for use. A user mentioned that even though they knew the negative consequences of smoking cigarettes, they ignored that knowledge and continued to smoke anyway (Table 3, Ex 37).

DISCUSSION

This study sought to capture the nuances of adolescents' perceptions of and motivations for using different tobacco products through eliciting personal narratives from both users and non-users about product appeal, social influences, and personal knowledge of the products. Examining themes that are similar and different between tobacco users and non-users is necessary for identifying factors that promote and deter adolescent use, with findings informative for targeted tobacco product prevention and intervention efforts. The findings from this qualitative study help bridge the gap in the literature that includes mostly quantitative analyses regarding youth preferences for each tobacco product, separately[10,25].

Previous quantitative studies have found that the social context of adolescents, both in school and among peer groups, influences perceptions and decisions to use tobacco products[26,27]. Adolescence is a dynamic and complex developmental phase during when youth are figuring out their personal identities and navigating through peer networks[28]. Participants' description of smoking as a part of community bonding supports existing research of the meaning and importance of these tobacco products for social empowerment and peer relationships[26]. Adolescents who have used tobacco noted that they initially did so because they were in a social situation or were introduced to a particular type of tobacco product by a peer. Users described being exposed to different types of tobacco in settings such as school sports teams, parties, and friends' houses, showing how the social environment can influence or enhance peer pressure on adolescents to experiment with and use tobacco. A participant also described how they were introduced to using tobacco products through an older romantic partner, which may parallel with failure to quit among adults who have a smoking spouse[29]. The social implications of these qualitative findings highlight the need for additional research and practice to inform campaigns and interventions that address tobacco use through social ties.

Participants described in detail why flavors were an appealing part of using tobacco products, with preferences for minty, sweet, and fruity flavors. Adolescents reported obtaining their tobacco, in particular e-cigarettes, through online and convenient

smoke shops where age verification is known not to be checked[30]. These findings emphasize the need for effective age verification and regulation of online sales[30,31].

The description of smoke tricks points to the possibility that adolescents are learning about them online through informal advertisements or videos, as opposed to flavors or youthful advertisements directly from tobacco companies[32]. These findings suggest the need to focus on both formal tobacco company-created marketing as well as informal advertising and social media in public health efforts aimed at regulating and reducing adolescent tobacco use.

Participants also reported a sense of social status and being "cool" using tobacco, especially as popularized by media, supporting research on how celebrities and 'pop culture' inflate the meaning and importance of different tobacco products for adolescents, and why more regulation of media representations of tobacco is needed[18,33]. Tobacco companies have a history of paying social media influencers to push tobacco marketing on channels frequented by youth, such as Instagram and YouTube[2,18]. Many users also described how they used tobacco simply to cope with boredom, and this could provide a potential avenue for tobacco prevention and intervention by keeping adolescents occupied with other appealing activities.

These qualitative results support existing research on how adolescents overwhelmingly perceive e-cigarettes as less harmful compared to combustible cigarettes, especially in regard to flavored products[6,8,12,19]. Many youth report that they would probably not smoke e-cigarettes and other alternative tobacco products without flavors[10,34]. Although mentioned less frequently throughout the interviews, some users also held the belief that cigars and smokeless tobacco were less harmful alternatives to combustible cigarettes, supporting existing qualitative and quantitative findings[17,19].

Strengths and limitations

Our study has some limitations. The sample size (25) was relatively small. Only ten participants had ever reported using tobacco products and we did not purposely sample among types of tobacco use. All participants were from Californian high schools so our results may not be generalizable to other adolescent populations in other areas with different local and state tobacco control policies. The interview guides for both users and non-users did not ask for the difference between tobacco versus nicotine, but instead discussed the products as one. Furthermore, the interview guides did not explicitly ask users to disclose each type of product they used, limiting the differentiation of themes across products. Interviews were conducted in 2015, prior to the surge in e-cigarette Juul use starting in 2017, and the tobacco product landscape has changed rapidly since then. Thus, perceptions of and decisions to use tobacco products among adolescents may differ between 2015 and now, especially given the implications of the COVID-19 pandemic[30]. However, Juul came on the market in 2015 and our early but timely interviews and the richness of our qualitative data are a valuable contribution to ascertain why Juul and other tobacco products, such as Puff Bar, rapidly increased in popularity with time.

CONCLUSIONS

Our findings suggest the need for healthcare providers, educators, and policymakers to understand why and how adolescents choose amongst different tobacco products, and are relevant to the current tobacco control landscape. Since the collection of the study data, local and state legislations have begun implementing flavor bans on tobacco products including e-cigarettes[35]. All flavors, including mint and menthol, should be regulated to deter appeal and aggressive marketing toward youth[10]. The easy access to the tobacco products reported by participants in this study supports the need to enforce the age limit laws for purchasing tobacco products online and in retail stores[36]. Our findings also highlight areas for future research and practice. Additional studies, both qualitative and quantitative, need to be carried out to examine adolescents' perceptions and use of different products within the same research study. Creative interventions around stress management and wellness can be designed to address some of the findings regarding why youth use certain tobacco products, especially for relaxation[37]. Robust school-based tobacco education should be supported to address adolescents' misperceptions, rather than expecting tobacco companies to inform adolescents of the harms of their new products[38,39]. Ultimately, it is important to empower adolescents and equip them with the

decision-making skills to better navigate away from situations in which product appeal, social influences, and conflicting information are encouraging them to use tobacco products.

REFERENCES

1. Johnston LD, Miech RA, O'Malley PM, Bachman JG, Schulenberg JE, Patrick ME. Monitoring the Future: National Survey Results on Drug Use, 1975-2018. 2018 Overview, key findings on adolescent drug use. Institute for Social Research, University of Michigan; 2019:126. http://www.monitoringthefuture.org/pubs/monographs/mtf-overview2018.pdf. Accessed November 15, 2020.
2. McCausland K, Maycock B, Leaver T, Jancey J. The Messages Presented in Electronic Cigarette–Related Social Media Promotions and Discussion: Scoping Review. J Med Internet Res. 2019;21(2):e11953. doi:10.2196/11953
3. Padon AA, Maloney EK, Cappella JN. Youth-Targeted E-cigarette Marketing in the US. Tob Regul Sci. 2017;3(1):95-101. doi:10.18001/TRS.3.1.9
4. McKelvey K, Halpern-Felsher B. From tobacco-endgame strategizing to Red Queen's race: The case of non-combustible tobacco products. Addict Behav. 2019;91:1-4. doi:10.1016/j.addbeh.2019.01.001
5. Roditis M, Delucchi K, Cash D, Halpern-Felsher B. Adolescents' Perceptions of Health Risks, Social Risks, and Benefits Differ Across Tobacco Products. J Adolesc Health. 2016;58(5):558-566. doi:10.1016/j.jadohealth.2016.01.012
6. McKelvey K, Baiocchi M, Halpern-Felsher B. Adolescents' and Young Adults' Use and Perceptions of Pod-Based Electronic Cigarettes. JAMA Netw Open. 2018;1(6):e183535. doi:10.1001/jamanetworkopen.2018.3535
7. Cooper M, Pacek LR, Guy MC, et al. Hookah Use Among US Youth: A Systematic Review of the Literature From 2009 to 2017. Nicotine Tob Res. 2019;21(12):1590-1599. doi:10.1093/ntr/nty135
8. Persoskie A, O'Brien EK, Nguyen AB, Tworek C. Measuring youth beliefs about the harms of e-cigarettes and smokeless tobacco compared to cigarettes. Addict Behav. 2017;70:7-13. doi:10.1016/j.addbeh.2017.01.033
9. Getachew B, Payne JB, Vu M, et al. Perceptions of alternative tobacco products, anti-tobacco media, and tobacco regulation among young adults: A qualitative study. Am J Health Behav. 2018;42(4):118-130. doi:10.5993/AJHB.42.4.11
10. Nguyen N, McKelvey K, Halpern-Felsher B. Popular Flavors Used in Alternative Tobacco Products Among Young Adults. J Adolesc Heal. 2019;65:306-308. doi:10.1016/j.jadohealth.2019.05.004
11. Bernat D, Gasquet N, Wilson KOD, Porter L, Choi K. Electronic Cigarette Harm and Benefit Perceptions and Use Among Youth. Am J Prev Med. 2018;55:361-367. doi:10.1016/j.amepre.2018.04.043
12. Amrock SM, Lee L, Weitzman M. Perceptions of e-Cigarettes and Noncigarette Tobacco Products Among US Youth. Pediatrics. 2016;138(5):e20154306. doi:10.1542/peds.2015-4306
13. Centers for Disease Control and Prevention. Outbreak of Lung Injury Associated with the Use of E-Cigarette, or Vaping, Products. https://www.cdc.gov/tobacco/basic_information/e-cigarettes/severe-lung-disease.html. Accessed August 2, 2020.
14. Leventhal AM, Strong DR, Kirkpatrick MG, et al. Association of electronic cigarette use with initiation of combustible tobacco product smoking in early adolescence. JAMA. 2015;314:700-707. doi:10.1001/jama.2015.8950
15. Jenssen BP, Boykan R. Electronic Cigarettes and Youth in the United States: A Call to Action (at the Local, National and Global Levels). Children (Basel). 2019;6(2):30. doi:10.3390/children6020030
16. Gaiha SM, Cheng J, Halpern-Felsher B. Association between youth smoking, e-cigarette use and COVID-19. J Adolesc Heal. 2020;67(4). doi:10.1016/j.jadohealth.2020.07.002
17. Cornacchione J, Wagoner KG, Wiseman KD, et al. Adolescent and Young Adult Perceptions of Hookah and Little Cigars/Cigarillos: Implications for Risk Messages. J Health Commun. 2016;21(7):818-825. doi:10.1080/10810730.2016.1177141
18. Phua J, Jin SV, Hahm JM. Celebrity-endorsed e-cigarette brand Instagram advertisements: Effects on young adults' attitudes towards e-cigarettes and smoking intentions. J Health Psychol. 2018;23:550-560. doi:10.1177/1359105317693912
19. Roditis ML, Halpern-Felsher B. Adolescents' Perceptions of Risks and Benefits of Conventional Cigarettes, E-cigarettes, and Marijuana: A Qualitative Analysis. J Adolesc Health. 2015;57:179-185. doi:10.1016/j.jadohealth.2015.04.002
20. Camenga DR, Fiellin LE, Pendergrass T, Miller E, Pentz MA, Hieftje K. Adolescents' perceptions of flavored tobacco products, including E-cigarettes: A qualitative study to inform FDA tobacco education efforts through videogames. Addict Behav. 2018;82:189-194. doi:10.1016/j.addbeh.2018.03.021
21. Kong G, Bold KW, Cavallo DA, Davis DR, Jackson A, Krishnan-Sarin S. Informing the development of adolescent e-cigarette cessation interventions: A qualitative study. Addict Behav. 2020;114:106720. doi:10.1016/j.addbeh.2020.106720
22. Liu J, McLaughlin S, Lazaro A, Halpern-Felsher B. What Does It Meme? A Qualitative Analysis of Adolescents' Perceptions of Tobacco and Marijuana Messaging. Public Health Rep. 2020;135(5). doi:10.1177/0033354920947399
23. SocioCultural Research Consultants LLC. Dedoose, Version 8.0.35: web application for analyzing qualitative and mixed methods research. www.dedoose.com.
24. Southwell BG, Niederdeppe J, Cappella JN, et al. Misinformation as a Misunderstood Challenge to

Public Health. Am J Prev Med. 2019;57(2):282-285. doi:10.1016/j.amepre.2019.03.009
25. Amrock SM, Gordon T, Zelikoff JT, Weitzman M. Hookah use among adolescents in the United States: Results of a national survey. Nicotine Tob Res. 2014;16:231-237. doi:10.1093/ntr/ntt160
26. Schaefer DR, Adams J, Haas SA. Social networks and smoking: exploring the effects of peer influence and smoker popularity through simulations. Health Educ Behav. 2013;40(1 Suppl):24S-32S. doi:10.1177/1090198113493091
27. Seo DC, Huang Y. Systematic review of social network analysis in adolescent cigarette smoking behavior. J Sch Health. 2012;82:21-27. doi:10.1111/j.1746-1561.2011.00663.x
28. Crosnoe R, Johnson MK. Research on Adolescence in the Twenty-First Century. Annu Rev Sociol. 2011;37:439-460. doi:10.1146/annurev-soc-081309-150008
29. Margolis R, Wright L. Better off alone than with a smoker: The influence of partner's smoking behavior in later life. J Gerontol B Psychol Sci Soc Sci. 2016;71:687-697. doi:10.1093/geronb/gbu220
30. Gaiha SM, Lempert LK, Halpern-Felsher B. Underage Youth and Young Adult e-Cigarette Use and Access Before and During the Coronavirus Disease 2019 Pandemic. JAMA Netw Open. 2020;3:e2027572. doi:10.1001/jamanetworkopen.2020.27572
31. Kong G, Morean ME, Cavallo DA, Camenga DR, Krishnan-Sarin S. Sources of electronic cigarette acquisition among adolescents in Connecticut. Tob Regul Sci. 2016;3:10-16. doi:10.18001/TRS.3.1.2
32. Clark EM, Jones CA, Williams JR, et al. Vaporous Marketing: Uncovering Pervasive Electronic Cigarette Advertisements on Twitter. PLoS One. 2016;11:e0157304. doi:10.1371/journal.pone.0157304
33. Mejia R, Mejia R, Morello P, et al. Movies promote tobacco use amongst adolescents: The need for policies to prevent this phenomenon. Rev Asoc Med Argent. 2018;131(1):24-31. PMID:30294003
34. Harrell M, Loukas A, Jackson C, Marti CN, Perry C. Flavored Tobacco Product Use among Youth and Young Adults: What if Flavors Didn't Exist? Tob Regul Sci. 2017;3:168-173. doi:10.18001/TRS.3.2.4
35. Friedman AS, Xu SQ. Associations of Flavored e-Cigarette Uptake With Subsequent Smoking Initiation and Cessation. JAMA Netw Open. 2020;3:e203826. doi:10.1001/jamanetworkopen.2020.3826
36. Tanski S, Emond J, Stanton C, et al. Youth Access to Tobacco Products in the United States: Findings From Wave 1 (2013-2014) of the Population Assessment of Tobacco and Health Study. Nicotine Tob Res. 2019;21:1695-1699. doi:10.1093/ntr/nty238
37. Holliday E, Gould TJ. Nicotine, adolescence, and stress: A review of how stress can modulate the negative consequences of adolescent nicotine abuse. Neurosci Biobehav Rev. 2016;65:173-184. doi:10.1016/j.neubiorev.2016.04.003
38. Liu J, Halpern-Felsher B. The Juul Curriculum Is Not the Jewel of Tobacco Prevention Education. J Adolesc Health. 2018;63(5):527-528. doi:10.1016/j.jadohealth.2018.08.005
39. Liu J, Gaiha SM, Halpern-Felsher B. A Breath of Knowledge: Overview of Current Adolescent E-cigarette Prevention and Cessation Programs. Curr Addict Rep. 2020;1-13. doi:10.1007/s40429-020-00345-5

CONFLICTS OF INTEREST

The authors have each completed and submitted an ICMJE form for disclosure of potential conflicts of interest. The authors declare that they have no competing interests, financial or otherwise, related to the current work. B. Halpern-Felsher reports grants from NIH, during the conduct of the study, that she is a paid expert in some e-cigarette litigation, and an unpaid scientific advisor and expert witness regarding some tobacco-related policies.

FUNDING

Research reported in this study was supported by grant number 1P50CA180890 from the National Cancer Institute and the Food and Drug Administration Center for Tobacco Products and grant number U54 HL147127 from the National Heart, Lung, and Blood Institute (NHLBI) and the Food and Drug Administration Center for Tobacco Products. The content is solely the responsibility of the authors and does not necessarily represent the official views of the NIH or the Food and Drug Administration.

AUTHORS' CONTRIBUTIONS

DR and BHF contributed to conceptualizing the study. DR and BHF contributed to the data collection. JL conducted the data analysis and drafted the initial manuscript. All authors contributed to the writing, reviewing and approval of the final manuscript. BHF is responsible for the overall content as guarantor.

PROVENANCE AND PEER REVIEW

Not commissioned; externally peer reviewed.
